# Influence of daily social stimulation on behavioral and physiological outcomes in an animal model of PTSD

**DOI:** 10.1002/brb3.458

**Published:** 2016-04-15

**Authors:** Shyam Seetharaman, Monika Fleshner, Collin R. Park, David M. Diamond

**Affiliations:** ^1^ Department of PsychologySt. Ambrose UniversityDavenportIowa52803; ^2^ Center for Preclinical and Clinical Research on PTSDUniversity of South FloridaTampaFlorida33620; ^3^ Department of Integrative Physiology and Center for NeuroscienceUniversity of ColoradoBoulderColorado80309; ^4^ Research & Development ServiceJames A. Haley VA HospitalTampaFlorida33612; ^5^ Department of PsychologyUniversity of South FloridaTampaFlorida33620; ^6^ Department of Molecular Pharmacology & PhysiologyUniversity of South FloridaTampaFlorida33620

**Keywords:** Posttraumatic stress disorder, psychiatric disorders, stress, trauma

## Abstract

**Introduction:**

We have shown in previous work that acute episodes of predator exposure occurring in the context of chronic social instability produced PTSD‐like sequelae in rats. Our animal model of PTSD contained two components: (1) *acute trauma*, immobilization of rats in close proximity to a cat twice in 10 days, and (2) *chronic social instability*, 31 days of randomized housing of cage cohorts. Here we tested the hypothesis that daily social stimulation would block the development of the PTSD‐like sequelae.

**Methods:**

Beginning 24 h after the first cat exposure, adult male rats were given our established PTSD model, alone or in conjunction with daily social stimulation, in which all rats within a group interacted in a large apparatus for 2 h each day for the final 30 days of the PTSD regimen. All *behavioral*, for example, anxiety, memory, startle testing, and *physiological assessments*, for example, body growth, organ weights, and corticosterone levels, took place following completion of the psychosocial stress period.

**Results:**

Daily social stimulation blocked the expression of a subset of PTSD‐like effects, including predator‐based cued fear conditioning, enhanced startle response, heightened anxiety on the elevated plus maze and the stress‐induced suppression of growth rate. We also found that social stimulation and psychosocial stress produced equivalent outcomes in some measures, including adrenal and heart hypertrophy, thymus atrophy, and a reduction in poststress corticosterone levels.

**Conclusions:**

Daily exposure of rats to a highly social environment blocked the development of a subset of trauma‐induced sequelae, particularly fear‐related outcomes. It is notable that daily social stimulation normalized a subset, but not all, of the PTSD‐like effects. We discuss our findings in the context of the literature demonstrating that social stimulation can counteract the adverse effects of traumatic stress on behavioral and physiological measures, as well as to produce its own stress‐like outcomes.

## Introduction

Individuals exposed to life‐threatening trauma involving intense fear, horror, and helplessness are at risk for developing posttraumatic stress disorder (PTSD; American Psychiatric Association, 1994). Our research team has developed a predator‐based psychosocial stress model of PTSD which has been shown to produce disturbances in behavior and physiology in rats which are analogous to the symptoms commonly reported in clinical PTSD (Stam 2007; Zoladz et al. 2008, 2012; Daskalakis and Yehuda 2014). Specifically, our group and others have demonstrated that acute predator exposure, with an obligate social instability component, produced PTSD‐like sequelae in rats, including a persistent traumatic memory, hypervigilance, heightened anxiety, memory impairment for new information, and neurotransmitter and hormonal abnormalities (Zoladz et al. 2008, 2012, 2013; Daskalakis et al. 2013; Wilson et al. 2013, 2014). Moreover, recent work from our group has shown that the PTSD‐like effects persisted for at least 4 months after cat exposure was initiated (Zoladz et al. 2015).

In clinical approaches to PTSD, one well‐documented risk factor is social support and stability, with increased rates of PTSD in combat veterans, victims of abuse, and survivors of natural disasters that report insufficient social support (Astin et al. 1993; King et al. 1998; Gold et al. 2000; Koenen et al. 2003; Ozer et al. 2003; Charuvastra and Cloitre 2008; Yehuda et al. 2015). Social support may be particularly effective in reducing PTSD risk in which both acute trauma (e.g., witnessing the mutilation of bodies) and chronic anxiety and stress (e.g., wartime combat) are experienced in the same context (Solomon et al. 1987).

Although associations between the social environment and PTSD are well described, identifying a causal link between the two is a challenge because of limitations inherent to human research. Research on animals administered environmental enrichment (EE) has provided convincing evidence that exposure of rats to highly social and complex environments, which typically involve a large number of animals interacting in an environment that contains novel objects and activity wheels, produces increased neocortical weight compared to conventional (impoverished) housing conditions (Diamond et al. 1964; West and Greenough 1972; Bennett et al. 1974; Diamond 2001). Research in this field has also shown that EE enhances hippocampal synaptic plasticity (Duffy et al. 2001; Leggio et al. 2005; Artola et al. 2006) and neurogenesis (Kempermann et al. 1997; Nilsson et al. 1999; Bruel‐Jungerman et al. 2005), and improves performance of rodents on spatial memory tasks (Teather et al. 2002; Leggio et al. 2005; Garrido et al. 2013). Furthermore, evidence indicates that EE is effective in preventing stress‐induced abnormalities in the brain and behavior. For example, EE blocked stress‐induced increases in fear and anxiety‐like behaviors (Roy et al. 2001; Benaroya‐Milshtein et al. 2004; Friske and Gammie 2005), impairment in spatial memory performance (Bredy et al. 2003; Wright and Conrad 2008), as well as impaired hippocampal synaptic plasticity (Yang et al. 2007; Hutchinson et al. 2012) produced by stress. Overall, these findings provide support for the hypothesis that EE increases the resilience of stressed rodents in response to traumatic stimuli (Fox et al. 2006). The robust effects of EE on improving stress outcomes are potentially of value in the study of animal models of PTSD. Therefore, our well‐established animal model of PTSD is of potential value in assessing the influence of EE on the expression of PTSD‐like outcomes in rats.

Previous work indicates that EE may enhance resiliency against stress‐induced changes in the brain and behavior through mechanistic pathways in common with antidepressant actions. For example, EE prevented stress‐induced neurochemical and morphological changes in the prefrontal cortex (Segovia et al. 2008, 2009) and hippocampus (Veena et al. 2009a,b), similar to antidepressant treatment (Tanti et al. 2013), supporting the hypothesis that EE acts in an antidepressant‐like manner to enhance stress resiliency, thereby facilitating recovery after stress exposure (Fleshner et al. 2011). The evidence of commonalities between antidepressant action and EE is relevant to recent work from our group. We demonstrated that daily administration of the antidepressant tianeptine blocked PTSD‐like changes produced in our animal model of PTSD (Zoladz et al. 2013). In that study, drug treatment was initiated 1 day after the first cat exposure and continued daily until the behavioral and physiological test battery was run. The approach in that work was to assess the effectiveness of pharmacotherapy which began 1 day after a trauma occurred to mimic a condition in which a person seeks treatment soon after a trauma occurs. In the current project, we followed this approach by initiating social stimulation 1 day after the first cat exposure occurred as a nonpharmacological form of posttrauma intervention.

Conventional EE procedures typically involve the inclusion of activity wheels in the rodents' home cages (Nilsson et al. 1999; Kempermann et al. 2002; Bruel‐Jungerman et al. 2005; Leggio et al. 2005; Artola et al. 2006; Yang et al. 2007; Hutchinson et al. 2012). However, the motor activity (exercise) component, alone, is a confounding variable, as exercise can enhance brain and behavioral indices of cognitive performance, and prevent, for example, the development of learned helplessness behaviors after uncontrollable stress (Gormezano and Prokasky 1987; van Praag et al. 1999; Greenwood and Fleshner 2011; Greenwood et al. 2011, 2013). Therefore, in the current work, we designed an environmental context which provided rats with the opportunity for extensive daily social interactions without an explicit exercise component. We hypothesized that daily social stimulation, based on a relatively brief (2 h/day) variation of EE overlapping in time with the psychosocial stress regimen, would ameliorate PTSD‐like effects in rats subjected to our chronic psychosocial stress regimen. Specifically, we tested the hypothesis that daily social stimulation would prevent the development of memory impairment, heightened anxiety, exaggerated startle, increased conditioned fear, as well as the physiological abnormalities (increased adrenal weight, decreased thymus weight, increased corticosterone levels) normally produced by chronic psychosocial stress.

Overall, our approach in using social stimulation to block the development of PTSD‐like effects in rats had two goals. First, it is well‐established that a greater level of social support is associated with reduced PTSD development in traumatized people. If this effect can be replicated in rats using a highly social environment, then the finding would strengthen the translational features of our PTSD model. Second, control over experimental variables in a rat model of PTSD offers the potential for assessing the mechanistic basis of how social stimulation is protective. Ultimately, this approach may provide insight into how trauma produces abnormalities in brain and behavior and at which level social interaction intervenes to normalize brain chemistry.

## Methods

### Animals and housing conditions

Adult male Sprague Dawley rats (225–250 g), which were not littermates, were obtained from Charles River. Upon arrival at the USF vivarium, the rats were housed in pairs on a 12:12‐h light–dark schedule (lights on at 0700 h) in Plexiglas cages (46 × 25 × 21 cm) with free access to food (Harlan Teklad Global 18% Protein Rodent Diet; Harlan Laboratories, Indianapolis, IN) and water. Rats were given 1 week to acclimate to the animal housing room following arrival before any experimental manipulations took place. After the acclimation period, all animals were randomly assigned to one of four groups (*n* = 8–10/group) based on a 2 × 2 experimental design. The two factors were (1) psychosocial stress model of PTSD (Stress) or no psychosocial stress (No Stress); and (2) daily social stimulation (Social) or no social stimulation (No Social). Therefore, the current study evaluated the following four groups: Social–Stress, No Social–Stress, Social–No Stress, No Social–No Stress. All procedures were in accordance with the University of South Florida's ethical guidelines on the treatment of animals in research.

### Psychosocial stress procedure

The psychosocial stress procedure followed here was based on the methodology described in detail previously (Zoladz et al. 2008, 2012). In brief, rats in the Stress groups were exposed to two acute predator stress sessions lasting 1 h each. The first session took place during the light cycle (between 0800 and 1500 h) and the second stress session occurred 10 days later during the dark cycle (between 1900 and 0200 h). In addition to the two cat exposures, rats in the Stress groups were subjected to unstable housing conditions (i.e., social instability) beginning on the day of the first cat exposure, and continued daily until the initiation of behavioral and physiological testing. Specifically, all of the rats within each Stress group were pseudorandomly paired with a different cage mate from within their group on a daily basis within existing (i.e., non‐neutral cages). The randomized housing began on the first day of cat exposure and continued until behavioral testing was initiated on Day 32.

Following procedures we have used previously (Zoladz et al. 2012, 2013, 2015), rats in the two Stress groups were administered a form of classical conditioning in which a neutral chamber was paired with immobilization and cat exposure. Specifically, on each of the 2 days of cat exposure each rat was first placed in a conventional fear conditioning chamber (25.5 × 30 × 20 cm; Coulbourn Instruments; Allentown, PA) for 3 min. A 30‐sec tone (74 dB @ 2400 Hz) was delivered during the last 30 sec of the exposure of the rat to the chamber. Following the 3 min chamber exposure, the rat was removed and immediately immobilized in a DecapiCone (Braintree Scientific, Braintree, MA) and then it was brought to the cat housing room, which was adjacent to the behavioral testing room.

In terms of classification of this form of classical conditioning, in pilot (unpublished) research, we have found that immobilization or cat exposure, alone, did not generate a conditioned fear response with re‐exposure of the rats to the chamber. Therefore, the unconditioned stimulus can be considered to be the combination of immobilization of the rat immediately upon its removal from the chamber, in conjunction with inescapable exposure to a cat, which took place approximately 45–60 sec later, after the rats were transported from the behavioral assessment room to the cat housing room. As immobilization was initiated immediately upon removal of the rat from the chamber, and then presumably the intensity of the US was intensified by cat exposure, this procedure may be considered a form of delay classical conditioning (Gormezano and Prokasky 1987).

In the cat housing room the rat was placed in one of 11 wedges of a circular Plexiglas pie‐shaped enclosure (20.5 × 20.5 × 8 cm/wedge; total pie diameter: 41 cm; Braintree Scientific), which was located inside of a large metal cage (61 × 53 × 51 cm). The metal cage contained an adult neutered female cat which remained on top of the pie‐shaped enclosure for the entire duration of the stress procedure. Soft fish‐flavored cat food was smeared on top of the pie‐shaped enclosure to direct the cat's motor and gustatory activity in the direction of the rats. Rats were subjected only to nontactile cues of the cat, as the pie‐shaped enclosure prevented physical contact between the rats and cat. Rats within a group were run sequentially, one at a time, until all rats were given the chamber and tone exposure followed by cat exposure for 1 h.

Rats in the No Stress groups were given the same handling and laboratory manipulations as the rats in the Stress groups, with the exception that they did not receive the stress manipulations (no immobilization, cat exposure, or daily social instability). Thus, the No Stress rats were brought to the laboratory on two occasions separated by 10 days and placed in the chamber for 3 min, with the tone delivered during the final 30 sec. At the end of the 3 min chamber exposure period the rats in the No Stress groups were returned to their home cages where they remained for 1 h, followed by their return to the vivarium. While rats in the Stress groups were housed in different pairs on a daily basis, rats in the No Stress groups were housed with the same cage cohort for the duration of the experiment.

### Daily social stimulation

The apparatus used as the social environment consisted of a chamber with three interconnected metal mesh levels which contained plastic tunnels, blocks, metal ladders, and a cloth hammock (91.44 × 63.50 × 157.48 cm; Ferret Nation, Muncie, IN). Beginning on Day 2, all animals in each of the two Social groups (Stress–Social and No Stress–Social) were transported from the housing room to the laboratory and placed into the social apparatus for 2 h each day, between 9 am and 1 pm. Each of the two Social groups was run at a different time. Specifically, all rats in the “Stress–Social” group were exposed to each other for 2 h/day and at another time, all rats in the “No Stress–Social” group were exposed to each other for 2 h/day.

The daily 2 h exposures continued from Day 2 until the first day of testing (Day 32). Between sessions, all objects were removed from the apparatus and cleaned with soap and tap water. Animals in the two No Social groups (Stress–No Social and No Stress–No Social) did not receive any exposure to the social apparatus. The rats in the No Social groups were transported to the laboratory following the timing of the Social groups, but they remained in their home cages for the 2 h period each day.

**Figure 1:**
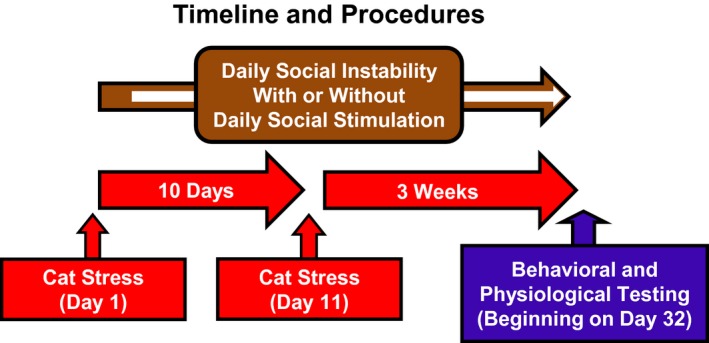
Timeline for psychosocial stress procedures and testing. Rats were exposed to the cat on Days 1 and 11. Beginning on Day 1, rats were also administered daily social instability, composed of pseudorandomized housing of each pair of rats in their home cages (brown arrow). Beginning on the day after the cat exposure (indicated by the white arrow within the brown arrow), rats in the social stimulation groups were brought to the laboratory and exposed to the social apparatus each day (Social) or they remained in their home cages (No Social). Social and stress manipulations terminated on Day 31, followed by the behavioral and physiological test battery, which began on Day 32.

An illustration of the timeline of all procedures is provided in Figure. 1.

### Behavioral testing

Three weeks after the second stress session, beginning on Day 32, all animals were administered a battery of behavioral and physiological tests which have been described in detail previously (Zoladz et al. 2008, 2012). Prior to the start of testing, the animals were handled individually for 2 min/day on three consecutive days. Body weights were recorded on the day of the initial stress session and on the first day of testing. On each behavioral testing day, rats were transported from the housing room into the laboratory and remained in their home cages for 30 min prior to the initiation of any behavioral tests.

### Fear‐conditioned memory testing

As described previously (Zoladz et al. 2012, 2013, 2015), on the first day of behavioral testing, predator‐based fear‐conditioned memory was measured as the percent of time rats spent immobile upon exposure to the context and cue which were paired with either the two cat exposures (Stress groups) or no cat exposures (No Stress groups). To assess contextual fear memory, rats were placed for 5 min in the same chamber which had been paired with the two cat exposures. One hour later, rats were tested for their memory of the cue (tone) presented in the chamber prior to either cat exposure (Stress groups) or no cat exposure (No Stress groups). During cue testing, rats were placed in a different chamber (25 × 22.5 × 33 cm; Coulbourn Instruments, Allentown, PA) than the one used during fear conditioning training. The first 3 min of cue testing was a baseline period without an auditory stimulus (tone), which was followed by 3 min with tone (74 dB @ 2400 Hz) delivery. Tones were presented through a speaker located on one side of the box; a light was on inside the chamber.

The cue test procedure provided a measure of motor activity of the rats in a novel context (pretone period). This 3‐min period also provided an assessment of general anxiety expressed to a place other than the context which was paired with cat exposure. Activity during delivery of the tone provided a measure of fear in response to the cue that had been presented just prior to immobilization and cat exposure in the Stress groups.

For both the contextual and cued fear memory tests, the number of fecal boli produced in the chambers were counted based on its utility as a biomarker of stress (Goldstein et al. 1996; O'Mahony et al. 2009). Immobility during fear testing was monitored by a 24‐cell infrared activity monitor (Coulbourn Instruments) mounted on the top of the boxes which used emitted infrared body heat images (1300 nm) from the animals to detect movement. Immobility was defined as continuous periods of inactivity, other than respiration, lasting ≥5 sec.

### Elevated plus maze

One day after the fear conditioning testing (Day 33), all rats were transported to the laboratory where they were tested on the elevated plus maze (EPM), which is a routine test of rodent anxiety (Korte and De Boer 2003). The apparatus (Hamilton‐Kinder; San Diego, CA) consists of two (11 × 51 cm) open and two closed (11 × 51 cm) arms which intersect to form the shape of a “plus” sign. Each rat was placed individually on the EPM for a period of 5 min where 64 infrared photobeams (located along the perimeter of the open and closed arms) linked to a computer program (Motor Monitor, Hamilton‐Kinder) scored a rat's location and activity. The computer program categorizes various behaviors for assessment, such as gross movement, time spent in each area of the apparatus, and head dips, in which rats extended their heads over the edge of the apparatus. The light intensity at the floor of the EPM was 5 Lux.

The primary dependent measure of anxiety was the percent of total time rats spent in the entire extent of the open arms of the EPM. Reduced time in the open arms is interpreted as increased anxiety. A secondary assessment of anxiety and exploratory, that is, risk taking, behaviors was the percent of time rats spent in the entrance (first third) compared to the end (last third) of the open arms of the EPM, which we refer to as “near” and “far” EPM, respectively. The secondary assessment in the open arms distinguished the time that rats spent at the entrance of the open arms (more anxiety, less exploration) compared to time rats spent traveling all the way to the end of the open arms (less anxiety/maximal exploration). In addition, the number of times a rat dipped its head below the side of the open arms (scored by the computer program each time rats' heads cross photobeam sensors along the edges and ends of the open arms) was recorded. Fecal boli were removed from the EPM between each of the 5 min testing sessions and the surface was cleaned using a 25% ethanol solution.

### Startle response

One hour after the EPM assessment, all rats were administered the acoustic startle response test. Each rat was placed inside a Plexiglas box (19 × 10 × 10 cm) which was on a sensory transducer, both of which were inside a larger cabinet (Hamilton‐Kinder; 36 × 28 × 50 cm). At the beginning of each session, rats were placed in the Plexiglas box. The sensory transducer was connected to a computer program (Startle Monitor; Hamilton‐Kinder), which recording the magnitude of the startle responses by measuring the amount of force (in Newtons) that rats exerted for a period of 250 msec after the presentation of each auditory stimulus. Differences in body weight were controlled for by adjusting the sensitivity settings on the sensory transducer (a range of 0–7 arbitrary units) prior to each session.

Each startle session began with a 5‐min acclimation (quiet) period, followed by the delivery of 24 white noise bursts (50 msec each) consisting of eight bursts at three different auditory intensities (90, 100, 110 dB) presented in sequential order. The time between each noise burst was pseudorandomly varied between 25 and 55 sec. After the start of the initial noise burst, the startle apparatus provided an uninterrupted background noise of 57 dB. Each session lasted approximately 17 min.

### Novel object recognition

Novel object recognition (NOR) is a commonly employed behavioral measure of memory (Broadbent et al. 2004; Hammond et al. 2004). One day after startle response testing, the rats were returned to the laboratory where they were placed in an open chamber. The apparatus consists of a plastic box with black walls and an open top (Hamilton‐Kinder; 40 × 47 × 70 cm). The rats spent 5 min in the chamber to habituate them to the environment prior to the training and testing sessions. All behaviors were monitored by a video feed to a computer program (Any Maze; Stoelting, Wood Dale, IL). The computer program provides for the assessment of behaviors exhibited in the open field, such as total distance travelled in each area (center and perimeter), total time spent in each area, and entries into each quadrant of the apparatus, which provide a source of assessment for the general behavior of the rats. One day after habituation to the open field, rats were placed in the same open field containing two identical (plastic/metal) objects for 5 min (training phase). The objects were placed in opposite, diagonally oriented corners of the open field and secured to the flooring with tape to prevent rats from manipulating and possibly displacing them.

The objects and their locations were counterbalanced across rats to control for place or object preferences. The testing phase commenced 3 h later in which rats were returned to the chamber, and one of the objects used during training was replaced by a novel object. A 16 cm^2^ zone was specified around each object to assess the time spent with the each object, and was measured by the computer program by tracking the rats' head movements in relation to the location of the object. During testing, greater time spent by rats in proximity to the novel versus familiar object was an indication of intact memory for the familiar object.

### Blood sampling, heart rate, blood pressure, and organ weights

Physiological testing commenced 1 day after the last day of behavioral testing. Blood samples were collected and then heart rate (HR) and blood pressure (BP) measurements were recorded from all rats to assess corticosterone (CORT) levels and cardiovascular responses, respectively. For baseline (undisturbed) blood sampling, rats were quickly transported, one cage at a time, from the housing room to an adjacent procedure room. A 0.5 cc sample of blood was collected from the saphenous vein within 2 min after the rats were removed from the housing room. The rats were then restrained in plastic DecapiCones (Braintree Scientific) for 20 min, followed by a second blood sample.

After the second blood sampling, the rats were placed in a Plexiglas tube (IITC Life Science, Woodland Hills, CA) within a warming test chamber (approximately 32°C) which served to facilitate blood flow to the tail. This procedure enabled HR and BP measurements to be taken using tail cuffs fitted with photoelectric sensors (IITC Life Science). The rats were then returned to their home cages for 1 h, after which a sample of trunk blood was collected via rapid decapitation. The adrenal glands, thymus glands, and hearts were then harvested and weighed. Blood samples were allowed to clot at room temperature, which were then centrifuged (3000 rpm) for 8 min, after which serum was extracted and stored at −80°C until assayed for CORT with an Enzyme ImmunoAssay kit from Assay Design Inc (cat # 901‐097, Ann Arbor, MI). All samples were diluted 1:50 and assayed per kit instructions.

### Statistical analyses

Unless otherwise noted, behavioral and physiological data were analyzed utilizing between‐subjects, two‐way ANOVAs with psychosocial stress (Stress, No Stress) and social environment (Social, No Social) serving as the between‐subjects factors. For the cue fear test, separate two‐way ANOVAs were used to analyze behavior before the tone (3 min) and during the final 3 min of testing where the tone was presented, respectively. The percent time in open arms in the EPM test was analyzed utilizing a between‐subjects two‐way ANCOVA, with psychosocial stress and social environment serving as the between‐subjects factors, and movement (ambulations) as the covariate. Startle responses at the three different acoustic stimuli intensity levels were analyzed separately utilizing within subjects two‐way ANOVAs. Planned comparisons (independent samples *t* tests) were conducted between groups that were predicted to differ a priori based on previous findings (Olsson et al. 1994; Yehuda et al. 1995a; Zoladz et al. 2008, 2012). For all statistical analyses, statistical significance was set at the *P *<* *0.05 level, and LSD post hoc tests were employed when appropriate.

## Results

### Contextual and cued fear expression

**Figure 2:**
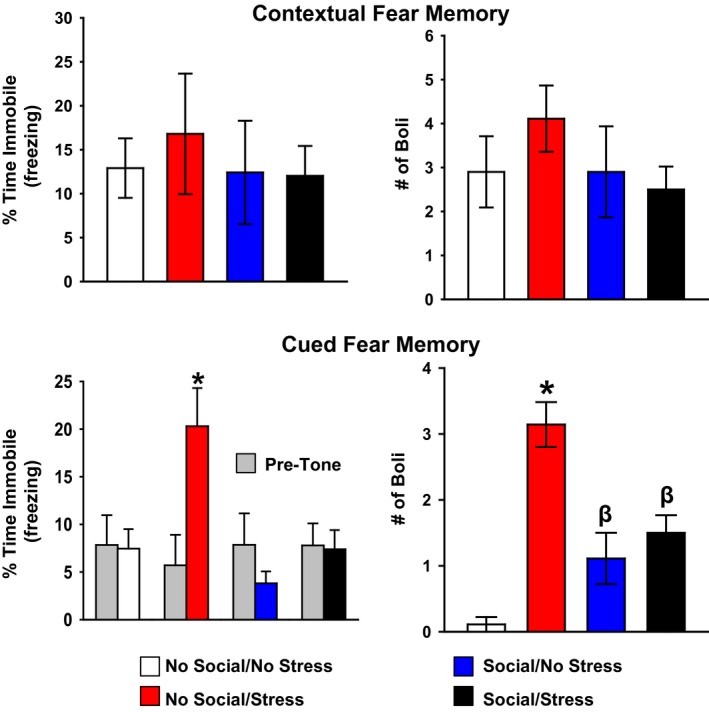
Assessment of contextual and cued predator‐based fear conditioning. There were no significant between‐group differences in immobility (upper left) or fecal boli production (upper right) upon re‐exposure of the rats to the cat‐associated context. There were significant between‐group differences in immobility (lower left) and fecal boli production (lower right) in response to the tone. Specifically, the No Social/Stress group showed a significant elevation in immobility relative to No Social/No Stress controls. This increase in immobility was prevented with social stimulation, indicated by significantly lower immobility levels in the Social/No Stress and Social/Stress groups. In a similar pattern, boli production was reduced with social stimulation relative to the No Social/Stress group. Note that both Social groups exhibited greater boli production than the No Social/No Stress group, and the No Social/Stress group produced the greatest number of boli of all groups (lower right). Data are presented as mean ± SEM. *P < 0.05 compared to all other groups; ^*β*^ *P* < 0.05 relative to the no social groups.

There were no significant between‐group differences in immobility to the context temporally paired with the two cat exposures (Fig. 2, upper left). Similarly, there were no significant main effects of either Stress or Social stimulation, and there was an absence of a Stress × Social stimulation interaction in the fecal boli analysis during contextual fear testing (Fig. 2, upper right).

For cued fear assessed during the 3‐min period prior to the tone, there were no significant main effects of Stress or Social and no significant Stress × Social interaction indicating that all four groups exhibited similar immobility levels prior to tone presentation (data not shown). During the tone, there was a main effect of Social, *F*(1, 28) = 8.99, *P* < 0.05, and a significant main effect of Stress, *F*(1, 28) = 8.84, *P* < 0.05. Planned comparisons revealed that, during the tone, the No Social/Stress groups showed greater immobility relative to No Social/No Stress control animals, *t*(13) = −2.45, *P* < 0.05. Additionally, this effect was blocked by social stimulation illustrated by the Social/Stress group showing significantly less immobility relative to No Social/Stress group, *t*(14) = −2.59, *P* < 0.05. The No Social/Stress group also showed significant elevations in immobility relative to Social/No Stress animals, *t*(13) = −3.35, *P* < 0.05 (Fig. 2, lower left).

The boli analysis during the cue fear test revealed a significant main effect of psychosocial stress, *F*(1, 29) = 33.55, *P *<* *0.05, and a significant Stress × Social interaction, *F*(1, 29) = 20.03, *P* < 0.05. Post hoc tests showed that the Stress/No Social group produced significantly more boli compared to all other groups, and both Social groups produced significantly more boli than both No Social groups (Fig. 2, lower right).

### Elevated plus maze

**Figure 3:**
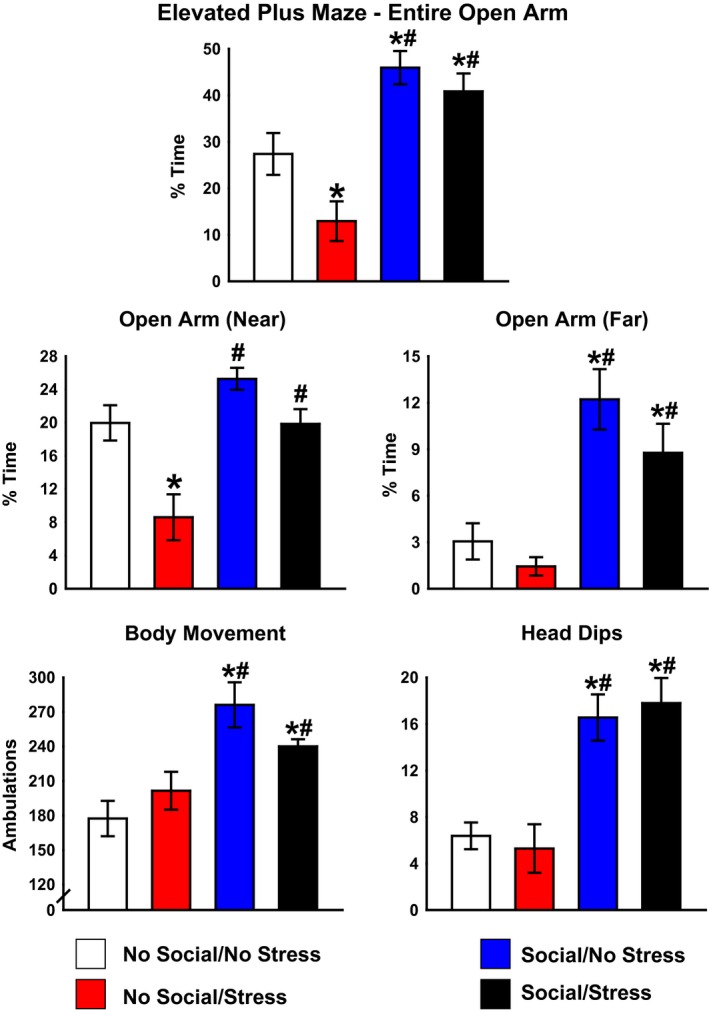
Activity in the elevated plus maze (EPM). Animals in the No Social–Stress group spent significant less time in the open arms of the apparatus, indicative of heightened general anxiety, which was prevented with social stimulation (top). The No Social–Stress group spent significantly less time in the near open arms of the EPM relative to the No Social–No Stress group. This effect was prevented with social stimulation (middle left). Both Social groups spent significantly more time in the far open arms of the EPM relative to both No Social groups (middle right). Both Social groups demonstrated significantly greater movement on the EPM relative to No Social controls (lower right). Social produced significantly more head dips on the EPM compared to the No Social groups (lower right). *P < 0.05 relative to No Social/No Stress. ^#^ *P* < 0.05 relative to Stress/No Social. Data are presented as mean ± SEM.

For EPM testing, there was a significant Stress × Social interaction, *F*(1, 26) = 4.21, *P *<* *0.05. Post hoc tests showed that the Stress/No Social group spent significantly less percent of time in the open arms compared to the No Stress/No Social and Stress/Social groups, indicating that daily social stimulation prevented the stress‐induced decrease in overall time spent in the open arms of the EPM. Both groups experiencing daily social stimulation showed significantly greater percent time spent in the open arms of the apparatus relative to both No Social groups (Fig. 3, top).

Assessment of time specifically in the entry area (near) versus the end (far) of the open arms of the EPM indicated a significant main effect of Stress, *F*(1, 30) = 14.60, *P *<* *0.05 and a significant main effect of social stimulation, *F*(1, 30) = 14.20, *P *<* *0.05. The Stress/No Social group spent significantly less time in the near open arms relative to No Social/No Stress group, and this effect was prevented in both social stimulation groups (Fig. 3, middle left). In the far open arms, there was a significant main effect for social stimulation, *F*(1, 31) = 23.39, *P *<* *0.05. Both Social groups exhibited greater time spent in the far open arms of the EPM relative to both No Social groups (Fig. 3, middle right).

Motor activity was assessed by examining overall ambulations made on the EPM. There was a significant main effect of social stimulation, *F*(1, 33) = 20.71, *P *<* *0.05, where both Social groups exhibited significantly more ambulations, indicative of increased motor activity on the EPM, relative to both No Social groups (Fig. 3, lower left). Analysis of head dips revealed a significant main effect of social stimulation, *F*(1, 30) = 35.33, *P *<* *0.05, indicating that both Social groups exhibited significantly more head dips on the EPM compared to both No Social stimulation groups (Fig. 3, lower right).

### Startle response

**Figure 4:**
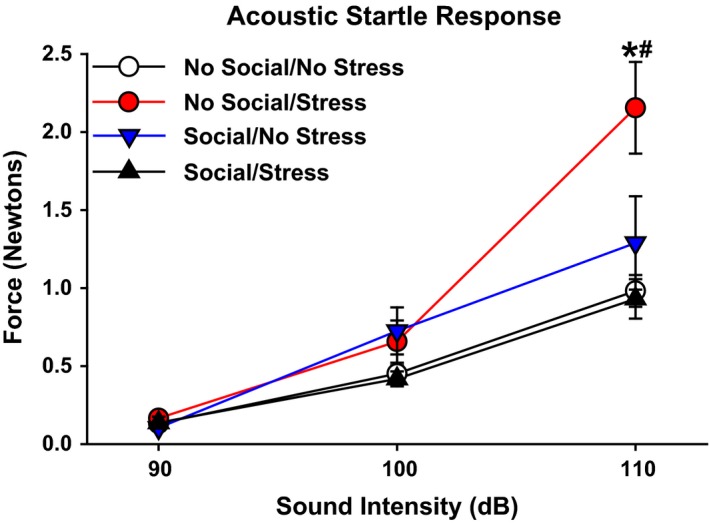
Startle response. There were no significant between‐group differences on acoustic startle response at the 90 and 100 dB stimulus intensity levels. At 110 dB, the No Social–Stress group exhibited a significant elevation in startle response relative to the other three groups. Data are presented as the mean startle response (Newtons) to the 90, 100, and 110 dB acoustic stimuli ± SEM. *P <* *0.05 relative to all other groups at 110 dB stimulus intensity.

There were no significant differences among groups on startle response magnitudes for the 90 and 100 dB acoustic stimuli. The responses to 110 dB revealed a significant main effect of social stimulation, *F*(1, 30) = 0.025, and a significant Stress × Social stimulation interaction, *F*(1, 28) = 12.76 (*P* < 0.05). Post hoc tests revealed that animals in the Stress/No Social group exhibited significantly greater startle responses than the other three groups at the 110 dB intensity (Fig. 4).

### Novel object memory

To assess performance on the NOR test, ratio times were calculated for each rat by dividing the time spent with the novel object by the time they spent with the familiar object for the 5 min testing period. Statistical analysis revealed no significant differences between No Stress/No Social (*M* = 1.53, SEM = 0.54), Stress/No Social (*M* = 1.07, SEM = 0.29), No Stress/Social (*M* = 0.80, SEM = 0.17), and Stress/Social (*M* = 1.87, SEM = 0.96). There were no significant main effects of either Stress, *F*(1, 28) = 0.50, or Social, *F*(1, 28) = 0.42, and the Stress × Social interaction was not significant, *F*(1, 28) = 0.85, all *P* values >0.05. Additional analyses, such as time with familiar and novel objects during the first minute, as well as time spent moving toward novel and familiar objects during the first and all 5 min of testing, and overall head distance from the novel and familiar objects, did not yield any significant differences among groups (data not shown). Overall, the methodological conditions for NOR testing rats in this study did not yield evidence of NOR memory in the control and stress groups.

### Corticosterone levels

**Figure 5:**
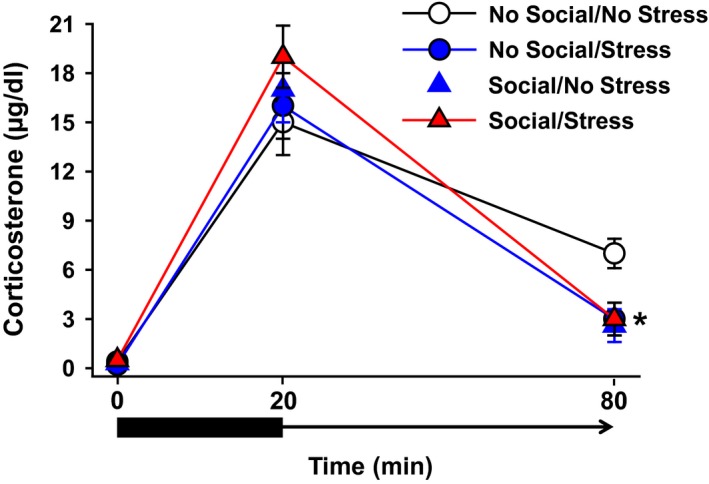
Serum levels of CORT under baseline, stress, and poststress conditions. There were no significant between‐group differences in CORT measures at baseline or after a 20‐min restraint stress period. At the 60‐min postrestraint time point, the No Social/Stress group exhibited significantly lower CORT levels than all three other groups. Data are shown as mean CORT (*μ*g/dL) ± SEM. *P < 0.05 relative to No Social/No PTSD.

Analysis of serum CORT levels revealed no significant main effects or interactions at any of the time points. Planned comparisons based on prior findings of enhanced negative feedback of animals housed under enriched environments (Mohammed et al. 1993) and in PTSD patients (Yehuda et al. 1995a), revealed that the control (No Stress/No Social) group exhibited significantly greater CORT levels at the 80 min time point relative to all three groups with Stress and/or Social stimulation manipulations (Fig. 5), *t*(14) = 2.70, social stimulation–no psychosocial stress, *t*(14) = 2.75, and social stimulation–psychosocial stress, *t*(13) = 2.65, *P* values < 0.05.

### Growth rate, organ weights, heart rate, and blood pressure

**Figure 6:**
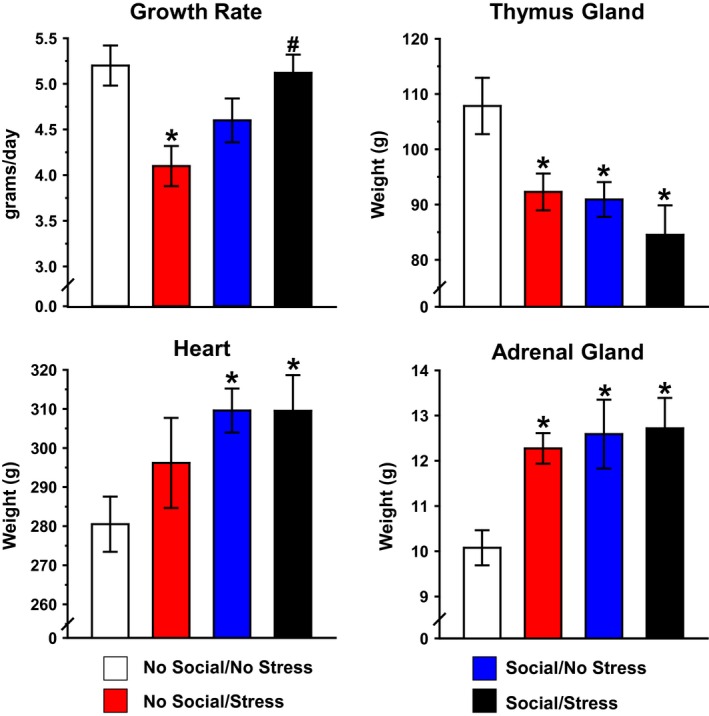
Influence of psychosocial stress on growth rate and organ weights. Psychosocial stress produced a significant decrease in growth rate. This effect was prevented in the Stress/Social group. Data are shown as mean g/day (upper left). Mean thymus gland weight was significantly lower in both Social groups and Stress/No Social relative to the No Social/No Stress group (upper right). Social stimulation produced a significant increase in mean heart weight relative to home cage controls (lower left). Both Social groups and No Social/Stress groups demonstrated robust increase in mean adrenal weight relative to home cage controls (lower right). Organ weights are shown as mean mg/100 g body weight. *P < 0.05 relative to No Social/No Stress. ^#^ *P* < 0.05 relative to Stress/No Social.

Growth rates were based on the increase in weight during the course of the 31‐day period of psychosocial stress. There was a significant Stress × Social stimulation interaction, *F*(1, 33) = 9.45, *P *<* *0.05. Post hoc tests showed a significantly reduced growth rate of the Stress/No Social group compared to No Stress/No Social and No Stress/Social groups (Fig. 6, upper left). The thymus gland exhibited the inverse effect of the adrenal gland in that there were significant main effects of Stress, *F*(1, 28) = 5.96, and social stimulation, *F*(1, 28) = 7.54 (*P*s < 0.05), and all three groups with Stress and/or Social factors produced a smaller thymus gland compared to the No Stress/No Social group (Fig. 6, upper right). There was a significant main effect of social stimulation for the heart weight, *F*(1, 32) = 4.79, *P *<* *0.05, and both Social groups exhibited greater heart weights than the No Social/No Stress group (Fig. 6, lower right). Analysis of adrenal weights revealed a significant main effect of social stimulation, *F*(1, 31) = 5.95, *P *<* *0.05, indicating that social stimulation produced significantly heavier adrenal glands than the no social manipulations. Post hoc tests showed that both social stimulation groups and the No Social/Stress group had significantly heavier adrenal glands compared to the No Social/No Stress group (Fig. 6, lower right). There were no significant effects of psychosocial stress or social stimulation on heart rate or blood pressure (data not shown).

## Discussion

We have investigated the influence of daily social stimulation on the expression of PTSD‐like effects in adult rats exposed to chronic psychosocial stress. The purpose in following this line of research was to assess the translational value of our PTSD model, as clinical studies have emphasized the role of inadequate social interactions as a risk factor for PTSD in civilians, as well as in military populations (Keane et al. 1985; Runtz and Schallow 1997; King et al. 1998; Koenen et al. 2003; Charuvastra and Cloitre 2008; Yehuda et al. 2015). We reported previously that predator exposure, alone, did not produce significant effects on behavior in our test battery; the expression of PTSD‐like effects required that the acute episodes of predator exposure occur in conjunction with daily social instability (Zoladz et al. 2008). This finding provided strong support for the hypothesis that for rats, as well as people, the social context is an important influence on whether acute trauma results in resilience or susceptibility to develop PTSD‐like effects (Daskalakis et al. 2013). In the current work, we extended our program on social factors as an influence on the expression of PTSD‐like effects in rats. Here we assessed the hypothesis that the inclusion of daily social interactions to the model would block the development of PTSD‐like effects on behavioral and physiological measures.

Our study has provided several noteworthy observations. First, we replicated findings from our PTSD model described previously, including reduced growth rate, reduced thymus weight, adrenal and cardiac hypertrophy, thymic atrophy, increased general anxiety‐like behavior (reduced open arm time in the elevated plus maze), increased acoustic startle response, and abnormalities in glucocorticoid (CORT) levels. Moreover, we replicated our finding that PTSD rats exhibited a long‐term (3 week) fear‐conditioned memory for a cue that occurred in close temporal proximity to immobilization and predator exposure (Zoladz et al. 2008, 2012, 2013, 2015). Second, and more specific to the issues in the current work, we have provided the novel observation that daily social stimulation blocked the expression of a subset of the PTSD‐like effects mentioned earlier. Third, in contrast to our expectations, social stimulation, alone, produced a subset of behavioral and physiological outcomes which are typically considered to be stress responses, including increased adrenal gland weight, increased heart weight, decreased thymus gland weight, and a lower poststress CORT level. Overall, this study has not only demonstrated that social stimulation can block a subset of adverse effects of traumatic stress, but it is also noteworthy that social stimulation, alone, produced stress‐like effects on behavior and physiology.

### Effects of social stimulation on traumatic memory expression

As the core feature of PTSD is a person's memory of a traumatic experience, we first assessed the effectiveness of social stimulation to ameliorate the expression of conditioned fear in the PTSD model. The measure of fear memory in animals is analogous to the fear expressed by people diagnosed with PTSD who formed a global memory of the trauma environment (context), as well as to more specific and distinct cues, such as sounds and visual cues which were associated with the trauma (Stam 2007). In our previous work, cat‐exposed rats exhibited a profound memory for both the context (fear conditioning chamber) and cue (the tone) that were temporally associated with immobilization and cat exposure (Zoladz et al. 2008, 2012, 2013, 2015). In the current work, however, cat‐exposed rats did not exhibit conditioned fear memory to the context (Fig. 2, top). One possible explanation for the absence of contextual fear memory expression was that cat exposure in the current study failed to evoke a fear response from the rats. This explanation is not tenable because the secondary measure of memory (conditioned cue memory), as well as other behavioral measures (discussed below), indicated that cat exposure generated a strong fear response in the rats.

An alternative explanation for the absence of a contextual fear memory was that a context‐specific extinction process developed in response to daily social stimulation, as well as to the daily transport to the laboratory all groups of rats experienced. That is, animals in all groups were transported from their housing room into the laboratory each day; rats in two of the groups were placed in the social apparatus (Social groups) and rats in the other two groups remained in their home cages in the laboratory (No Social groups). This procedure was a departure from all of our previous work as daily transport to the laboratory had not been a part of our psychosocial stress regimen. We hypothesize that daily transportation to the laboratory environment, which included placement of all rats in a room near the fear conditioning chamber, served as a form of extinction for the context which was temporally associated with cat exposure.

In contrast to the extinction of contextual fear memory, there was a maintenance of the cued fear memory in the group that received the PTSD regimen without social stimulation. That is, the No Social/Stress group exhibited the greatest magnitude of cue‐evoked immobility (Fig. 2, left) and fecal boli production (Fig. 2, right) of all four groups. We hypothesize that the uniqueness of the cue associated with cat exposure, and its absence when rats were transported to the laboratory each day, enabled the conditioned fear cue memory to be resistant to the extinction process produced by daily transport of the rats to the laboratory.

The maintenance of the cued component of the conditioned fear memory, and its resistance to extinction from repeated returns to the general vicinity of the conditioning chamber, is potentially of value toward understanding PTSD memory dynamics. That is, investigators have theorized as to why traumatic memories are different from less emotional memories. In particular, van der Kolk and colleagues have described how traumatic memories are “fragments” of the original experience, with a pathologically intense focusing on isolated cues associated with the trauma, with impaired recollection of the overall context of the experience (van der Kolk and Fisler 1995). Similarly, Ehlers et al. (2002) described the focusing of memory processing on cues that had occurred proximal to trauma onset as the “warning signal hypothesis” which describe how isolated cues take precedence in trauma memory processing. Indeed, therapy for trauma has been described as a “contextualization” process whereby the isolated cues become incorporated into a broader context to facilitate trauma recovery process (Ehlers and Clark 2000; Liberzon and Sripada 2008). Our unexpected finding of a selective extinction of the contextual fear memory and maintenance of cued fear memory in the No Social/Stress group may be of value in identifying the neurobiological processes (i.e., hippocampal vs. amygdala) underlying the differential expression of contextual versus cued fear memory in traumatized people who develop PTSD (Rougemont‐Bucking et al. 2011; Linnman et al. 2012; Pitman et al. 2012).

It is noteworthy that the cue memory fear expression was specific to the cue, itself, and not a result of a generalized fear to the novelty of the cue testing environment. Hence, there was no effect of stress on immobility when the rats were exposed to the novel environment, prior to cue delivery (gray bars in Fig. 2, lower left). While immobility in the precue period was equivalent across groups, delivery of the cue generated a significant increase in immobility only for the No Social/Stress group. This finding reinforces the value of our approach to isolate the long‐term (3 week) persistence of the tone‐signaled (predator‐based) fear memory.

The cued fear memory findings from the No Social/Stress group were a dramatic contrast to the findings from the Social/Stress group. Inclusion of daily social stimulation to the PTSD model completely abolished the expression of cued fear memory, as indicated by the absence of freezing (immobility) in the Social/Stress group in response to cue delivery (Fig. 2, lower left). Therefore, one may conclude that a socialization process suppressed the cue‐evoked fear memory. These results are consistent with other research indicating that EE is effective in reducing fear expression (freezing) when animals were presented with a cue associated with shock (Benaroya‐Milshtein et al. 2004) and predator exposure (Klein et al. 1994).

Overall, there are two notable features of our work on fear conditioning and social stimulation. First, we have demonstrated that the contextual component of traumatic memory processing was more susceptible to extinction than the cued fear memory. This finding is potentially relevant toward understanding the hippocampal (context) versus amygdala (cued) components of trauma memory processing (Pitman et al. 2012). Second, the finding that social stimulation blocked the expression of cued fear memory in rats indicates that social stimulation may lower the risk of clinical PTSD because it blunts the expression of conditioned fear in response to cues which were associated with trauma.

### Social stimulation (and environmental enrichment) as a form of chronic stress

In the previous section we discussed how daily social interactions blocked the expression of cued fear memory. This finding is consistent with the view that social stimulation, as with EE, appears to be protective against the adverse effects of chronic stress. The stress‐reversing effects of EE has led other investigators to refer to EE as the “functional opposite of stress” (Fox et al. 2006; Wright and Conrad 2008; Solinas et al. 2010). However, there is the intriguing and counterintuitive finding of significant boli production by both Social groups in the cue chamber compared to the No Social/No Stress group (Fig. 2, lower right). The equivalent increase in boli production by both Social groups was not driven by an association of the cat with the cue because the Social/No Stress group had not been exposed to the cat, and yet this group produced increased boli production in the novel cued environment. The increased boli production by both Social groups paradoxically suggests that animals with greater social experience responded with greater anxiety‐like behavior when they were exposed to a novel environment.

Although unexpected, the finding of increased apparent anxiety with social stimulation is consistent with research demonstrating that EE rats subsequently exposed to a mildly stressful novel environment exhibit greater boli production compared to impoverished controls (Green et al. 2010). Thus, although increased boli production is normally attributed to a rodent expression of heightened anxiety, the EE literature and our work indicate that, for reasons that are not fully understood, rats with greater social experience generate greater boli production in response to novelty.

It is noteworthy that a subset of other measures which are commonly considered stress responses were increased in our social groups, as well. For example, we found that the Stress and Social groups both developed adrenal gland and heart hypertrophy, thymus gland atrophy, and a reduction of poststress corticosterone levels (Figs. 5, 6). It is important to note that, even though animals in the social stimulation groups were not provided with running wheels, the tri‐level apparatus did provide the animals an opportunity for more physical activity than they would have experienced in their home cages. The possibility that social stimulation induced an increase in physical activity which contributed to the organ weight changes is consistent with findings that exercise alone can produce adrenal hypertrophy and thymic atrophy (Anderson et al. 2002; Naylor et al. 2005). In addition, we found that social stimulation increased heart weight relative to the No Stress/No Social group, which may have been driven by greater physical activity in the social groups (Moraska et al. 2000).

Our findings are relevant to an analysis of stress‐like effects on EE in a recent review (Crofton et al. 2015). These authors stated that EE (and by extension, social stimulation), should not be considered the “functional opposite of stress.” Instead, they observed that a high degree of social interactions appears to serve as a mild form of chronic stress that inoculates animals (and people) from the ravages of traumatic stress. Thus, extensive daily social interactions appear to act as a mild form of “healthy” stress which enhances resilience in the face of traumatic situations (Fox et al. 2006), and yet, produces stress‐like effects, as well. Overall, our findings help to distinguish components of the behavioral stress measures that are specific to trauma, for example, cue‐specific fear memory, open arm avoidance in the EPM, increased startle response, and reduced growth rate, from those that are produced by the mild stress components of extensive social stimulation.

### Relation to studies on environmental enrichment

Our work can be considered to be an extension of research which has demonstrated beneficial effects of EE on behavioral and physiological measures. Specifically, our findings extend EE research which has largely focused on the benefits of 24 h EE housing on brain and behavioral enhancement (Nilsson et al. 1999; Duffy et al. 2001; Bruel‐Jungerman et al. 2005; Leggio et al. 2005), and recovery from stress‐induced brain and behavioral impairments (Bredy et al. 2003; Fox et al. 2006; Yang et al. 2007; Wright and Conrad 2008). In the current experiment, we showed that social stimulation, composed of acute (2 h) daily exposures during a month of psychosocial stress, prevented a range of behavioral responses specifically related to PTSD.

The development of fear and anxiety‐like behavior may be driven by similar neurobiological mechanisms. For instance, previous work indicates that chronic stress produces dendritic retraction in the mPFC (Radley et al. 2004). In contrast, EE has been found to increase mPFC dendritic spine density (Kolb et al. 2003). In addition, EE‐induced resiliency to heightened anxiety and depressive‐like behavior was prevented with infralimbic cortex lesions in the PFC (Lehmann and Herkenham 2011). It is possible, therefore, that social stimulation blocked stress‐induced increases in fear and anxiety‐like behavior through an enhancement of mPFC functioning.

Using similar methods to our psychosocial stress model of PTSD, Wilson and colleagues assessed neurochemical changes in the PFC. These authors demonstrated that PTSD rats exhibited significant reductions in PFC serotonin (5‐HT) levels (Wilson et al. 2014), whereby others have indicated EE‐induced increases in PFC 5‐HT (Brenes et al. 2008). In like fashion, rats treated with the antidepressant buspirone (a 5‐H1TA agonist) for 3 weeks showed increased hippocampal 5‐HT1A mRNA levels (Chen et al. 1995), similar to increases produced by EE (Rasmuson et al. 1998). Related work has demonstrated that stress‐induced increases in the dopaminergic and cholinergic systems are prevented under enrichment conditions (Segovia et al. 2008, 2009), similar to studies using antidepressant treatment (Dazzi et al. 2001). In addition, the current findings of an attenuation of PTSD‐like effects with social stimulation are similar to our previous work demonstrating that the antidepressant tianeptine, which attenuates glutamatergic activity, blocked all stress effects in our PTSD model (Zoladz et al. 2013). Therefore, social stimulation may have produced alterations in neurochemical systems in a manner comparable to those produced by antidepressant treatment, a hypothesis which is supported by findings that EE produces antidepressant‐like changes (Fox et al. 2006; Hendriksen et al. 2012). This hypothesis is further supported by our results indicating that rats administered daily social stimulation exhibited increased head dips, on the EPM, an index of exploratory‐like behavior (Wall and Messier 2001). This is suggestive of an antidepressant‐like effect given previous results indicating an increase in head dips produced by acute antidepressant drug treatment (Griebel et al. 1997; Silva and Brandao 2000).

In addition to behavior, we tested for the effects of psychosocial stress and social stimulation on physiological responses. Our primary physiological dependent measure was the level of corticosterone (CORT), which is elevated under stress conditions, as a result of hypothalamic–pituitary–adrenal (HPA) axis activation. CORT contributes to a negative feedback loop on the HPA axis by binding to glucocorticoid receptors (GRs) in the hypothalamus and pituitary gland. In PTSD, patients show increased number of GRs (Yehuda et al. 1995a), supporting research indicating attenuated CORT levels shortly after trauma, such as in victims of rape (Resnick et al. 1995) and motor vehicle accidents (Delahanty et al. 2000). Interestingly, similar to observations in PTSD, EE animals exhibited an upregulation of GR mRNA expression in the hippocampus (Mohammed et al. 1993; Olsson et al. 1994), which may serve to facilitate more efficient negative feedback control, thereby blunting the CORT response.

We found that social stimulation produced a reduced poststress CORT response relative to NoSocial–Stress group. This finding extends work demonstrating that EE can produce a reduction in poststress levels of CORT (Moncek et al. 2004; Hutchinson et al. 2012). This finding suggests that the social procedure enhanced recovery from stress‐induced impairments in behavior, in part, by mimicking physiological stress responses (Crofton et al. 2015).

Our CORT findings complement results indicating that Stress rats pretreated with the synthetic glucocorticoid dexamethasone (DEX) displayed a more rapid recovery of postimmobilization CORT levels relative to nonstressed controls (Zoladz et al. 2012). In theory, social stimulation increased CORT levels at the time of stress mimicking DEX effects, and, as a result, enhanced CORT recovery following immobilization, perhaps due to a greater number or sensitivity of GRs. The current work, therefore, provides guidance as to neuroendocrine approaches to follow in subsequent work. Additionally, the organ weight results corroborate the CORT finding in that social stimulation produced similar profiles to that of stress. Specifically, chronic psychosocial stress produced a significant increase in adrenal gland weight, decreased thymus weight and decreased growth rate, replicating previous work by our group (Zoladz et al. 2008, 2012) and others (Wilson et al. 2014). Interestingly, social stimulation produced a similar organ weight pattern to stress which supports previous research indicating heavier adrenals (Bakos et al. 2004), more activity in the adrenal cortex (Marashi et al. 2003) and thymic atrophy (Tsai et al. 2002) in EE animals. These findings are, again, indicative that aspects of social stimulation mimic the physiological stress response, perhaps as a coping mechanism.

### Absence of effects of PTSD and social manipulations on a subset of measures

In contrast to our findings of PTSD effects in multiple measures of anxiety and physiology, we did not find any significant evidence of memory impairment produced by the chronic psychosocial stress model nor any memory enhancement produced by social stimulation. This was surprising given the substantial evidence indicating that EE enhances hippocampal long‐term potentiation (LTP; Duffy et al. 2001; Artola et al. 2006), neurogenesis (van Praag et al. 2000; Brown et al. 2003; Bruel‐Jungerman et al. 2005), neurotrophic growth factors (Mohammed et al. 1993), dendritic length (Rosenzweig and Bennett 1996; Faherty et al. 2003; Leggio et al. 2005), and performance on hippocampus‐dependent tasks (Nilsson et al. 1999; Ickes et al. 2000; Leggio et al. 2005). This result was also unexpected given recent findings indicating that EE prevented stress‐induced impairment in hippocampal integrity and spatial memory performance (Ickes et al. 2000; Hutchinson et al. 2012).

It is important to consider methodological factors to understand why performance on the NOR task was poor for all groups. The NOR literature suggests that relatively long delays (>90 min) between training and memory testing in the NOR task are associated with greater dependence of memory retrieval on hippocampal, as well as perirhinal cortical, functioning (Clark et al. 2000; Baker and Kim 2002; Broadbent et al. 2004; Hammond et al. 2004; Balderas et al. 2008; Winters et al. 2008). Therefore, we deployed a 3‐h delay period between training and testing which, in theory, would be sufficiently challenging to reveal any PTSD‐like memory impairment (Yehuda et al. 1995b; Bremner 2006; Samuelson 2011). However, in the current work, as in a previous study from our group (Zoladz et al. 2015), there was no evidence of intact NOR memory with the 3‐h delay period in any group. An explanation for the absence of NOR memory is that NOR training and testing occurred after the animals had already been assessed for fear‐conditioned memory, anxiety‐like behavior, startle response and habituation to the open field. It is therefore likely that the poor NOR memory performance was the result of experimental “noise” stemming from the multiple days of prior testing. In addition, it is likely that only 1 day of exposure of the rats to the testing chamber prior to training was an insufficient period of time for the rats to habituate to the chamber prior to the training and memory test day. In more recent work we have found that 3 days of habituation to the testing environment prior to the explicit training day facilitates the expression of novel object memory (unpublished data). Therefore, in subsequent work we will minimize preliminary behavioral testing when we include NOR training and memory testing, as well as to include a multiday period of habituation of rats to the testing chamber prior to NOR training.

With regard to cardiovascular measures, there were no significant differences between any groups on heart rate (HR) or blood pressure (BP). This finding is inconsistent with previous work from our group in which psychosocial stress significantly increased systolic and diastolic BP and lowered HR in one study (Zoladz et al. 2008), and elevated HR in another (Zoladz et al. 2013). We would postulate that the absence of significant effects on cardiovascular responses here may be due to differences in methods between the current experiment and the previous work. In the current study, animals in all groups were transported from the housing room into the laboratory daily. This daily transport experience in the current work, compared with no daily transport in the previous studies, could have influenced the outcomes on cardiovascular measures, as well as contextual fear memory (discussed previously). Additionally, the human literature reveals inconsistencies with regards to HR and BP in PTSD, with differences depending on factors such as length of diagnosis, time of day and comorbidities (Zoladz and Diamond 2013). Therefore, the absence of PTSD‐like effects on cardiovascular outcomes here is not completely unexpected, and may reflect the complexity of how social stimulation interacts with acute trauma to influence cardiovascular responses.

### Limitations and areas for future research

In designing this study, we had theorized that daily social stimulation would serve to ameliorate chronic psychosocial stress‐induced responses, in part, by neutralizing the social instability component of the paradigm. That is, prior work has demonstrated that social instability is a critical component of our psychosocial stress paradigm to produce PTSD‐like effects in rats (Zoladz et al. 2008). We hypothesized that, just as in human clinical conditions, exposing rats to a group of conspecifics on a daily basis would attenuate the anxiety‐provoking features of social instability in their home cages. Moreover, the anxiety‐provoking features of daily social instability in the home cages were further attenuated because the same rats were used in daily social instability as well as in daily social stimulation. Thus, although the cohort pair for each rat changed on a daily basis, the rats had the opportunity to experience all rats in the stress group on a daily basis. The great value of this finding is that the rodent work reinforces the view of social support and stability as a critical component in the recovery from trauma in people.

To relate our findings more closely to previous work (Hutchinson et al. 2012), additional research may provide a clearer understanding of the protective influence of social stimulation when it is initiated prior to the onset of chronic stress. Even though we aimed to control for factors outside of social stimulation, future studies should aim to tease out possible differences produced by other subcomponents inherent in the social stimulation procedure. For example, isolating the potential influence of the effects of physical activity from social stimulation, per se, on the observed outcomes is important given that social stimulation animals did show greater movement on the EPM. However, we would suggest that the increased motor activity of social versus no social groups is not a confounding factor in the interpretation of EPM behavior. Most importantly, the location within the apparatus where more activity occurred is more important than their overall motor activity. The rats in both social groups chose to spend a greater percentage of their time in the open arms than both no social groups. Moreover, the social groups explored the entire length of the open arms, exploring the end of the open arms, with more open arm head dips, than the no social groups. This finding indicates that the socializing process appears to have emboldened the rats to be more exploratory, that is, less fearful even in the Stress group, than no social rats. Our findings are also consistent with related findings in which EE rats exhibited greater motor activity, more head dips and overall greater exploration of open areas on a variant of the elevated plus (the elevated zero maze) (Sampedro‐Piquero et al. 2013).

Finally, it is important to address physical from social factors in modifying behavioral and physiological measures. Physical activity, alone, has been shown to prevent stress‐induced decreases in hippocampal BDNF (Adlard and Cotman 2004), ameliorate the persistence of conditioned fear (Greenwood et al. 2007), and also to result in CORT and hippocampal GR levels similar to those produced by stress (Fediuc et al. 2006). These findings point to the need of further investigation into the specific role which physical activity, as compared to social activity, per se, may have played in the effects reported in the current study.

From a translational perspective, clinical studies show a strong relationship between social support and lower rates of PTSD and related symptoms. The literature suggests that the size of one's social network may not be as important as the quality of support received (Cohen and Wills 1985). The assessment of the quality of social relationships in human research is based on an individual's perceptions of the nature of their interactions. Utilizing an animal model is useful in that the opportunities for social interactions can be experimentally manipulated, with potential study of positive, as well as negative, effects on PTSD development. Therefore, it must be noted that the current study is an investigation of social stimulation in the form of increased opportunity for interaction in a larger social network relative to a more stagnant home cage control condition.

Future studies should quantify the social interactions in our animal model. For instance, subsequent work could measure the frequency and duration of interactions of the rats to potentially relate their behavior to relationship factors in human social situations. In the current study conclusions based on the quality of support cannot be drawn since we did not quantify their interactions; it was evident, however, from our incidental observations that in the social environment there was near constant activity and interactions among the rats. It should be acknowledged, however, that relationship quality and social support are difficult to assess, in part, because of limitations which are inherent in comparing animal and human research (van Erp et al. 1994).

## Summary and Conclusions

This is the first work we are aware of to show empirical evidence that a daily social intervention is effective in preventing PTSD‐like responses in rodents. Specifically, we found that a 2‐h period of social stimulation blocked the chronic psychosocial stress generated cue fear memory, anxiety‐like behavior, startle response, and reduced growth rate. In addition, we found that social stimulation mimicked aspects of stress, which may contribute to an increased resilience of the stress response system to subsequent stressors. Our results contribute to the literature by providing translational evidence consistent with the finding that social support confers resistance of traumatized people to develop PTSD. This level of analysis in an animal model serves to underlie the importance of clinical research addressing social factors which mitigate risk factors for PTSD, as well as nonpharmacological treatments for the disorder.

## Conflict of Interest

None declared.
